# Online Health Misinformation Susceptibility Increases Health Risk Behaviors and Vaccine Hesitancy: Evidence from Greece

**DOI:** 10.3390/healthcare14040425

**Published:** 2026-02-08

**Authors:** Ioannis Moisoglou, Aglaia Katsiroumpa, Olympia Konstantakopoulou, Aris Yfantis, Olga Galani, Maria Tsiachri, Panagiota Peleka, Zoe Katsiroumpa, Petros Galanis

**Affiliations:** 1 Department of Nursing, University of Thessaly, 41500 Larissa, Greece; iomoysoglou@uth.gr; 2 Clinical Epidemiology Laboratory, Faculty of Nursing, National and Kapodistrian University of Athens, 11527 Athens, Greece; aglaiakat@nurs.uoa.gr (A.K.); mtsiachri@nurs.uoa.gr (M.T.); gpeleka@nurs.uoa.gr (P.P.); zkatsiroumpa@nurs.uoa.gr (Z.K.); pegalan@nurs.uoa.gr (P.G.); 3 Center for Health Services Management and Evaluation, Faculty of Nursing, National and Kapodistrian University of Athens, 11527 Athens, Greece; 4 Special Vocational Education Laboratory of Fthiotis, Directorate of Secondary Education of Fthiotis, 35100 Lamia, Greece; arisyfantis@gmail.com; 5 Faculty of Nursing, University of West Attica, 12243 Athens, Greece; ogalani@uniwa.gr

**Keywords:** health misinformation, susceptibility, health behaviors, vaccine hesitancy, risk, fake news

## Abstract

**Background**: Health-related misinformation is a pervasive phenomenon that expanded substantially during the COVID-19 pandemic, particularly regarding concerns about vaccine safety and effectiveness. **Objective**: The aim of this study was to investigate the impact of online health misinformation susceptibility on health behaviors and vaccine hesitancy. **Methods**: A cross-sectional study with a convenience sample of 402 individuals was conducted in Greece, with data collected via an online survey during September 2025. We used the Health-Related Online Misinformation Susceptibility Scale to measure online health misinformation susceptibility. The Health Behavior Inventory–Short Form was used to measure health behaviors, while the Vaccine Hesitancy Scale (VHS) was used to measure participants’ hesitancy towards vaccination. We performed multivariable analysis to identify the independent effect of health misinformation after adjustment for several confounders. **Results**: Multivariable linear regression analysis showed a positive association between online health misinformation susceptibility and diet score (adjusted coefficient beta = 0.026; 95% confidence interval [CI] = 0.006 to 0.046; *p* = 0.010) and anger and stress score (adjusted coefficient beta = 0.033; 95% CI = 0.013 to 0.052; *p* = 0.001). After adjustment for confounders, we found a positive association between online health misinformation susceptibility and score on the factors “lack of confidence” (adjusted coefficient beta = 0.016; 95% CI = 0.005 to 0.028; *p* = 0.006) and “risk perception” (adjusted coefficient beta = 0.023; 95% CI = 0.010 to 0.036; *p* = 0.001). **Conclusions**: Our findings suggest that higher susceptibility to online health misinformation is associated with poorer health behaviors and greater vaccine hesitancy.

## 1. Introduction

Citizens increasingly seek online information on a wide range of topics, among which health-related information constitutes a prominent area of interest. Such information-seeking behavior is not confined to the websites of healthcare or health-related organizations (e.g., hospitals or healthcare professionals) but also extends to social networking platforms. The scope of online health information is broad and encompasses general health information, specific diseases, medications and treatments, nutrition and physical activity, medical resources, disease-related symptoms, health promotion, as well as health-related news and policies [1,2]. Nevertheless, in many instances, the information received by users is not scientifically substantiated in accordance with current scientific evidence; however, it is often perceived as scientifically valid and useful, and is frequently reproduced and disseminated further through social media platforms [3,4]. False information that is shared without the intention to mislead or to cause harm is defined as misinformation [5]. Access to the web, combined with the ease of information sharing through social media platforms, facilitates the rapid dissemination of misinformation.

We are now living in an era in which misinformation can spread with great ease. This is largely attributable to citizens’ widespread and readily available access to social media platforms, where misleading content can be repeatedly reproduced and circulated across multiple channels. Sustained exposure to misinformation increases individuals’ susceptibility to believing such content and to sharing it with others. This phenomenon is commonly referred to as the “illusory truth” effect [6]. Such forms of misinformation have targeted vaccines, arguably the most important tool for safeguarding public health, as well as health-related behaviors that may place citizens at risk.

Vaccines have historically been the target of opposition primarily from the anti-vaccination movement, which has raised concerns regarding their safety and effectiveness. These concerns have included allegations that vaccines may cause brain damage, seizures, intellectual disability, and autism. As a consequence, childhood vaccination coverage declined, leading to the resurgence of infectious diseases [7]. This phenomenon of vaccine skepticism was particularly pronounced during the COVID-19 pandemic, during which widespread misinformation circulated regarding medical issues, vaccine development processes, and conspiracy theories, ultimately exerting a negative influence on vaccination intention [8,9,10].

Misinformation also affects other domains of health-related behaviors, including substance use and smoking, as well as dietary habits. Specifically, with regard to smoking and illicit drug use, misinformation primarily concerns the purported safety of e-cigarettes and their alleged positive role in smoking cessation, as well as claims regarding the safe and effective use of drugs in the absence of scientific evidence [11,12]. Also, online nutrition-related information is often inaccurate and of low quality [13]. Misinformation related to nutrition may pertain to claims concerning weight loss, as well as to purported beneficial effects of specific foods on the prevention and treatment of chronic diseases [14,15,16]. Fake news and misinformation can also exert a significant impact on mental health, affecting both individuals who disseminate such content and those who are exposed to it [17,18].

The phenomenon of misinformation is also strongly present in Greece, influencing citizens’ willingness to opt for vaccination. In particular, health misinformation in Greece, propagated through social media, messaging apps, and occasionally mainstream outlets, influences vaccination primarily by weakening trust in public institutions and amplifying perceived vaccine risks [19]. In the first COVID-19 lockdown, a national survey found that only 57.7% of adults reported willingness to be vaccinated, and willingness was associated with higher COVID-19 knowledge and rejection of conspiracy claims such as laboratory-origin narratives [20]. Greek studies also link conspiratorial thinking (e.g., “hidden agenda” beliefs) with higher hesitancy [21], while a 2025 network analysis suggests that, among hesitant individuals, misinformation is tightly connected to beliefs about vaccine safety/effectiveness and to distrust in authorities, creating self-reinforcing belief systems [22]. Evidence from health professionals indicates that obtaining vaccine information from official public health authorities is strongly associated with greater acceptance, highlighting the protective role of trusted institutional communication; national data on healthcare staff similarly map information pathways across television and formal health bodies [23]. Vaccine hesitancy is further reinforced by the anti-vaccination movement, which in Greece has been particularly active online and across social media platforms. During the COVID-19 pandemic, this movement reflected a blend of distrust toward institutional actors and concerns regarding the safety and potential adverse effects of COVID-19 vaccination [24].

The characteristics of individuals who are vulnerable to the consumption of misinformation include higher levels of perceptual bias, increased depressive symptoms, and lower educational attainment. In particular, individuals with higher levels of perceptual bias, more depressive symptoms, and lower educational have higher likelihood of consuming misinformation [25]. Furthermore, individuals who report high levels of distrust, particularly toward governmental institutions, are more likely to be misinformed [26]. Also, people with lower trust in scientists and those who believe in conspiracy behaviors show higher levels of misinformation susceptibility [27]. Especially with regard to vulnerability to health-related misinformation, identified predictive factors include health-related anxiety, pre-existing misinformation beliefs, and repeated exposure to health misinformation, as well as female gender and lower socioeconomic status [28]. Among nurses, higher levels of trust in scientists, possession of an MSc or PhD degree, and expressed interest in politics were associated with reduced susceptibility to online health misinformation [29].

Despite increasing recognition that online health misinformation constitutes a significant threat to public health, empirical research has largely concentrated on exposure to misinformation or on evaluating fact-checking. In this context, the literature on the consequences of online health misinformation susceptibility is scarce. To date, no published research has systematically measured individuals’ susceptibility to online health misinformation as a distinct construct within Greece, nor explored its potential influence across a broader set of maladaptive health behaviors. This represents a notable gap, given Greece’s unique sociocultural context, patterns of institutional trust, and digital literacy challenges, which may shape how individuals evaluate and act upon health information online. The present study addresses this gap by providing the first empirical assessment of online health misinformation susceptibility in a Greek population and by examining how this susceptibility is associated with both vaccine hesitancy and multiple health risk behaviors. By operationalizing susceptibility directly, rather than relying on proxy variables, the study offers a more precise understanding of how individuals process health information in digital environments. Furthermore, by linking susceptibility to a range of real-world behaviors—such as avoidance of medical guidance, use of unverified remedies, and other risk-enhancing practices—the findings extend prior literature. As such, this study contributes novel evidence relevant to public health communication in Greece and provides a conceptual and methodological foundation for future comparative and intervention-oriented research.

In this context, this study sought to investigate the impact of online health misinformation susceptibility on health behaviors and vaccine hesitancy.

## 2. Materials and Methods

### 2.1. Study Design

A cross-sectional study was conducted in Greece, with data collected via an online survey during September 2025. The questionnaire was developed using Google Forms and disseminated through social media platforms, including Facebook, Instagram, and LinkedIn, yielding a convenience sample. We posted the study questionnaire in four public pages on Facebook, in four public pages on Instagram, and in two public pages on LinkedIn. Eligibility criteria required participants to (1) be adults, (2) spend at least 30 min daily on the web or social media to ensure a minimum exposure to online information, (3) provide informed consent, (4) understand the Greek language since we performed the study in Greece, and (5) have access to the internet. On the other hand, we excluded from our study illiterate populations, children under 18 years old, and non-Greek speakers. Since we performed our study in Greece, we used Greek versions of the study questionnaires, and thus we excluded non-Greek speakers to avoid information bias. The study adhered to the Strengthening the Reporting of Observational Studies in Epidemiology (STROBE) guidelines [30].

It should be noted that the existing literature on the associations among susceptibility to online health misinformation, health risk behaviors, and vaccine hesitancy is limited. Consequently, it remains unclear whether susceptibility to online health misinformation precedes the development of health risk behaviors and vaccine hesitancy, or whether these variables influence susceptibility instead. Furthermore, the cross-sectional design of our study does not allow for the establishment of causal relationships among these three variables. Thus, the directionality of the associations between susceptibility to online health misinformation, health risk behaviors, and vaccine hesitancy cannot be determined. Although some epidemiological studies draw upon well-established theoretical frameworks or biological mechanisms to support causal pathways [31,32], such foundations are not available for the variables examined in our study.

Also, we should describe in brief the pros and cons of convenience sampling using digitally launched surveys [33,34,35]. Digitally distributed surveys require minimal financial and logistical resources compared with traditional face-to-face or telephone methods. Thus, we can rapidly collect large amounts of data without printing materials, travel, or hiring field staff. Online platforms enable participants to respond immediately, allowing researchers to gather substantial sample sizes in short timeframes. For instance, individuals may complete an online study questionnaire that was shared by others in their social media platforms, thus enabling the snowball sampling method. Also, digital environments (e.g., social media groups, online forums, and health-related communities) allow researchers to reach participants who may be geographically dispersed or difficult to recruit through traditional methods. Moreover, respondents can complete the survey at their own pace and in their preferred environment, which may increase response rates and reduce respondent burden. On the other hand, in the digitally launched surveys, participants self-select into the study based on interest, web access, and platform use. This leads to samples that may not represent the target population. For example, individuals with limited digital literacy or web access may be underrepresented, while frequent social media users may be overrepresented. Moreover, because the sample is not random and may differ systematically from the broader population, findings cannot be confidently generalized. Results are more suitable for exploratory insights than population-level inference. Also, convenience sampling in digital environments lacks a defined sampling frame, making it impossible to calculate response rates accurately or estimate sampling error. Overall, although digitally launched convenience sampling offers practical advantages in terms of cost, speed, and accessibility, its inherent limitations related to selection bias and reduced generalizability must be carefully acknowledged when interpreting study findings.

Sample size was calculated using G*Power v.3.1.9.2. With one predictor and six confounders included in the multivariable models, an anticipated effect size of 0.04 for predictor–outcome association, a statistical power of 95%, and a 5% type I error, the required sample size was estimated at 327 participants.

### 2.2. Measurements

Data were collected on participants’ demographic characteristics, including gender (male or female), age (analyzed as a continuous variable), and educational attainment (elementary school, middle school, high school, or university degree). Financial status was measured using a self-assessment scale ranging from 0 (very poor) to 10 (excellent). Participants also self-rated their digital literacy on a scale from 0 (very poor) to 10 (very good) and reported the average number of hours spent per day using the web and/or social media, which was treated as a continuous variable.

We used the Health-Related Online Misinformation Susceptibility Scale (HR-OMISS) [36] to measure online health misinformation susceptibility in our sample. The HR-OMISS is an adapted version of the Online Misinformation Susceptibility Scale (OMISS) [37] that measures online misinformation susceptibility in general, while the HR-OMISS measures specifically online health misinformation susceptibility. The HR-OMISS includes nine items and answers are on a five-point Likert scale: never (5), rarely (4), sometimes (3), very often (2), and always (1). Total score ranges from 9 to 45. Higher scores indicate higher misinformation susceptibility. We used the valid Greek version of the HR-OMISS [36]. In our study, Cronbach’s alpha for the HR-OMISS was 0.883. Our data supported the unidimensional structure of the HR-OMISS since we performed confirmatory factor analysis (CFA) and we found that x^2^/df was 2.231 (acceptable value is less than 5), root mean square error of approximation (RMSEA) was 0.055 (acceptable value is less than 0.10), goodness of fit index (GFI) was 0.977 (acceptable value is higher than 0.90), adjusted goodness of fit index (AGFI) was 0.946 (acceptable value is higher than 0.90), Tucker–Lewis index (TLI) was 0.974 (acceptable value is higher than 0.90), incremental fit index (IFI) was 0.986 (acceptable value is higher than 0.90), normed fit index (NFI) was 0.975 (acceptable value is higher than 0.90), and comparative fit index (CFI) was 0.986 (acceptable value is higher than 0.90).

The Health Behavior Inventory–Short Form (HBI-SF) [38] was used to measure health behaviors among our participants. The HBI-SF includes 12 items and answers are on a 7-point Likert scale from 1 (strongly disagree) to 7 (strongly agree). Six items are reverse-scored so that higher scores on all items indicate a greater degree of health risk. The HBI-SF includes four factors; diet (three items); proper use of health care resources (three items); anger and stress (three items); and substance use (three items). The score on each factor is obtained by summing answers and dividing by the number of items. Ultimately, the score on each factor ranges from 1 to 7, with higher values indicating higher level of health risk behaviors. We used the valid Greek version of the HBI-SF [39]. In our study, Cronbach’s alpha for the four factors of the HBI-SF ranged from 0.713 to 0.824. Our data supported the four-factor structure of the HBI-SF since we performed CFA and we found that x^2^/df was 1.387, RMSEA was 0.031, GFI was 0.977, AGFI was 0.956, TLI was 0.953, IFI was 0.972, NFI was 0.907, and CFI was 0.971.

The Vaccine Hesitancy Scale (VHS) [40] was used to measure participants’ hesitancy towards vaccination. The VHS includes 10 items and answers are on a 5-point Likert scale from 1 (strongly disagree) to 5 (strongly agree). Seven items are reverse-scored so that higher scores on all items indicate higher levels of vaccine hesitancy. The VHS includes two factors: lack of confidence (seven items) and risk perception (three items). The score on each factor is obtained by summing answers and dividing by the number of items. Ultimately, the score on each factor ranges from 1 to 5. We used the valid Greek version of the VHS [41]. In our study, Cronbach’s alpha for the factors “lack of confidence” and “risk perception” was 0.917 and 0.704, respectively. Our data supported the two-factor structure of the VHS since we performed CFA and we found that x^2^/df was 3.169, RMSEA was 0.074, GFI was 0.971, AGFI was 0.920, TLI was 0.963, IFI was 0.984, NFI was 0.976, and CFI was 0.983.

### 2.3. Ethical Issues

The study protocol received formal approval from the Ethics Committee of the Faculty of Nursing at the National and Kapodistrian University of Athens (approval No. 75, 13 July 2025). The research complied with the ethical principles outlined in the Declaration of Helsinki [42]. Data collection was conducted anonymously and on a voluntary basis, following a comprehensive briefing on the study’s objectives and procedures, after which participants provided informed consent.

### 2.4. Statistical Analysis

Categorical variables were summarized as frequencies and percentages, whereas continuous variables were expressed as means with standard deviations (SD) and medians with interquartile ranges. Assessment of normality was performed using the Kolmogorov–Smirnov test and Q-Q plots, confirming that continuous variables exhibited a normal distribution. Our independent variable was online health misinformation susceptibility, while the dependent variables included health behaviors and vaccine hesitancy. Given the normal distribution of the dependent variables, linear regression analysis was performed. Initially, simple linear regression analysis was conducted to examine univariate associations, followed by a multivariable regression analysis to estimate the independent effect of online health misinformation susceptibility. The results are presented as unadjusted and adjusted beta coefficients, along with 95% confidence intervals (CI) and *p*-values. We performed the Breusch–Pagan test to examine heteroskedasticity in the multivariable linear regression models. *p*-values higher than 0.05 in the Breusch–Pagan test indicate absence of heteroskedasticity, and, thus, models are acceptable. Also, we calculated Variance Inflation Factor for the independent factors to examine collinearity in the multivariable linear regression models. Variance Inflation Factors greater than 5 suggest serious collinearity. The Breusch–Pagan statistics and Variance Inflation Factors in our multivariable models were acceptable (see Section 3). Additionally, Pearson correlation coefficients were calculated to evaluate associations between normally distributed scale scores. Statistical significance was set at *p* < 0.05. All analyses were performed using IBM SPSS Statistics for Windows, version 28.0 (IBM Corp., Armonk, NY, USA).

## 3. Results

### 3.1. Demographic Characteristics

The study sample consisted of 402 participants, of whom 76.6% were female. The mean age was 48.74 years (SD = 9.52), with a median age of 50 years and an interquartile range of 10 years. The average financial status score was 5.60 (SD = 1.45), while the median score was 6 (interquartile range = 2). Digital literacy exhibited a mean score of 7.49 (SD = 1.90) and a median of 8 (interquartile range = 3). On average, participants reported spending 3 h per day on web/social media platforms (SD = 2.36), with a median of 2 h and an interquartile range of 2.5 h. Table 1 shows the demographic details of our participants.

### 3.2. Study Scales

The mean score on the HR-OMISS scale was 22.28. Since the range of the HR-OMISS scale is from 9 to 45, the mean score of 22.28 in our study indicates a moderate level of health-related online misinformation susceptibility among our participants. In relation to health-related behaviors, the average scores for the dimensions “diet”, “appropriate utilization of health care resources”, “anger and stress management”, and “substance use” were 2.94, 2.64, 4.10, and 1.58, respectively. Regarding vaccine hesitancy, the mean score for lack of confidence was 3.92, whereas the mean score for risk perception was 3.06. A detailed summary of descriptive statistics for all study measures is presented in Table 2.

Table 3 summarizes the correlations among the study variables. Online health misinformation susceptibility showed positive correlations with diet score (r = 0.144; *p* = 0.004), anger and stress score (r = 0.147; *p* = 0.003), lack of confidence score (r = 0.119; *p* = 0.017), and risk perception score (r = 0.199; *p* < 0.001). These findings indicate that higher susceptibility to online health misinformation was associated with poorer health behaviors and greater vaccine hesitancy.

### 3.3. Dependent Variable: Health Behaviors

Table 4 shows the results from the linear regression analysis with score on the Health Behavior Inventory–Short Form as the dependent variable. Multivariable linear regression analysis showed a positive association between online health misinformation susceptibility and diet score (adjusted coefficient beta = 0.026; 95% CI = 0.006 to 0.046; *p* = 0.010) and anger and stress score (adjusted coefficient beta = 0.033; 95% CI = 0.013 to 0.052; *p* = 0.001). In other words, a 1-unit increase in the HR-OMISS (range of scale: 9 to 45) was associated with a 0.026 increase in the diet score (range of scale: 1 to 7) and a 0.033 increase in the anger and stress score (range of scale: 1 to 7). We did not find statistically significant associations between online health misinformation susceptibility and scores on the factors “proper use of health care resources” and “substance use”.

### 3.4. Dependent Variable: Vaccine Hesitancy

Table 5 shows the results from the linear regression analysis with score on the Vaccine Hesitancy Scale as the dependent variable. After adjustment for confounders, we found a positive association between online health misinformation susceptibility and score on the factors “lack of confidence” (adjusted coefficient beta = 0.016; 95% CI = 0.005 to 0.028; *p* = 0.006) and “risk perception” (adjusted coefficient beta = 0.023; 95% CI = 0.010 to 0.036; *p* = 0.001). In other words, a 1-unit increase in the HR-OMISS (range of scale: 9 to 45) was associated with a 0.016 increase in the factor “lack of confidence” (range of scale: 1 to 5) and a 0.023 increase in the factor “risk perception” (range of scale: 1 to 5).

## 4. Discussion

The present study showed a statistically significant association between susceptibility to online health misinformation and poorer health behaviors, as well as increased vaccine hesitancy. However, we should notice that the effect of online health misinformation susceptibility on health behaviors and vaccine hesitancy was poor since the adjusted coefficient beta ranged from 0.016 to 0.033, while the model R^2^ ranged from 3.5 to 6.1%. These small values for model R^2^ indicate that the online health misinformation susceptibility interprets only a small percentage of the variation in health behaviors and vaccine hesitancy in our study. Therefore, there is a need to identify more variables that could explain poor health behaviors and vaccine hesitancy in the general population. Similarly, correlation coefficients indicated small correlations between variables since they ranged from 0.119 to 0.199.

Regarding vaccine hesitancy, our findings are consistent with those reported in the existing literature [8]. Vaccination has historically been accompanied by a degree of skepticism, particularly concerning vaccine safety, a phenomenon that is further amplified when a novel vaccine is introduced, as was the case with the COVID-19 vaccines [43]. Unsubstantiated concerns about vaccine safety, lacking scientific evidence, are frequently propagated by the anti-vaccination movement. By exploiting social media platforms, this movement disseminates misinformation and fake news, which are easily and rapidly amplified and reproduced [44]. The more frequently a false claim is repeated and disseminated, the greater the likelihood that individuals will come to accept it as true [45]. In the domain of health-related information, repeated exposure to health misinformation actively fosters its acceptance [28] and may contribute to the development of vaccine hesitancy [46]. The tendency for individuals to become vulnerable to misinformation and the mechanisms underpinning this vulnerability are commonly conceptualized as the “illusory truth” effect [47]. Empirical evidence indicates that repetition increases perceived veracity, such that statements encountered multiple times are judged as more truthful than unfamiliar statements. Because misinformation is often amplified through repeated circulation across mainstream media, political communication, and influencer-driven social platforms, the “illusory truth” effect has become increasingly salient in contemporary information environments [45]. The main cognitive mechanism underlying people’s tendency to judge repeated statements as true is processing fluency: as a claim is reiterated, it becomes more familiar and consequently easier to mentally process. Put differently, the mind often treats this sense of ease as a heuristic cue for truthfulness. Importantly, empirical evidence indicates that (1) prior exposure to misinformation can increase its perceived accuracy; (2) the “illusory truth” effect emerges for both plausible and implausible assertions; (3) existing knowledge does not reliably shield individuals from this bias; and (4) the effect does not seem to depend strongly on thinking style, showing little modulation by whether reasoning is more analytical or more intuitive [45].

Since vaccine hesitancy has not only individual-level consequences but also significant implications for public health, it is essential to first investigate the factors that may affect health misinformation hesitancy, as well as identifying the population groups that are more susceptible to accepting health-related misinformation. Individuals with lower educational attainment, members of minority populations, those with limited health literacy, people who exhibit distrust toward the healthcare system, individuals holding positive attitudes toward alternative medicine, persons with specific political orientations, and those with limited analytical thinking skills are more susceptible to health misinformation [48,49,50]. Healthcare professionals constitute a key determinant in mitigating susceptibility to health misinformation, particularly regarding vaccine acceptance. Empirical evidence indicates that individuals who place greater trust in scientists and healthcare professionals demonstrate lower susceptibility to health-related misinformation and exhibit a higher likelihood of accepting vaccination [48,51]. Consequently, healthcare professionals should possess the requisite knowledge and skills to respond effectively to the questions and concerns of citizens and patients, and to be able to critically challenge and counteract misinformation that may have been erroneously adopted as scientifically substantiated.

Regarding health-related behaviors, the present study showed a statistically significant association between online misinformation susceptibility and dietary practices (adjusted coefficient beta = 0.026; R^2^ = 4.0%), as well as elevated levels of anger and stress (adjusted coefficient beta = 0.033; R^2^ = 5.2%). Misinformation regarding food and nutrition is particularly prevalent on social media platforms. Such content frequently includes claims about “miracle diets” and broader forms of dietary misinformation related to specific pathologies, including unfounded assertions concerning disease prevention or treatment, as well as the enhancement of immune system function [52]. Individuals with higher levels of misinformation are more likely to disseminate inaccurate food safety information online [53]. The source of nutrition-related information plays a crucial role, with individuals who place trust in nutrition professionals being more likely to adopt higher-quality dietary patterns [54].

The multifaceted impacts of misinformation render it imperative that citizens possess the appropriate skills to critically appraise both the credibility of online sources from which they seek health-related information and the validity of content disseminated through social media platforms. Experimental studies have showed that the acquisition of skills related to literacy concepts, including information, news, media, and digital literacies, significantly enhances participants’ ability to identify fake news and misinformation [55,56,57,58]. These programs should be designed to be applicable to both school pupils and university students, as they are not only direct consumers of misinformation, but also citizens who, throughout their lives, will frequently seek health-related information through online sources [59,60]. Recently in Greece, the Minister of Education announced the pilot implementation of educational programs for lower and upper secondary school students aimed at strengthening their skills in identifying fake news [61].

The present study has several limitations. First, its cross-sectional design precludes the establishment of causal relationships among the examined variables. In addition, the demographic characteristics of the participants could have included further variables, such as occupation, prior exposure to misinformation, and training in misinformation recognition. Consequently, future longitudinal studies could explore the potential confounding role of these variables to establish a more valid association between online health misinformation susceptibility and health behaviors and vaccine hesitancy. Moreover, we used self-reported instruments to measure online health misinformation susceptibility, health behaviors and vaccine hesitancy and therefore information bias is probable in our study. Although valid instruments were employed to assess the study variables, participants may have responded in a socially desirable manner, potentially leading to an underestimation of misinformation susceptibility, an overreporting of favorable health behaviors, and a reduced indication of vaccine hesitancy. Additionally, our convenience sample through an online survey does not allow us to generalize our findings in the general population of Greece. Our sample cannot be representative of the general population in Greece since the educational level of our participants was very high, and also most of them were females. Probably, our highly educated sample could underestimate online misinformation susceptibility and vaccine hesitancy. On the other hand, the high level of education among our participants could lead to an overestimation of digital literacy. Thus, future research with random, stratified and representative samples is essential to improve our findings regarding the association between online health misinformation susceptibility, health behaviors and vaccine hesitancy. In this context, research in other countries with different cultural settings will add significant evidence to this research topic. Previous vaccine behavior is another important variable that can affect the association between online health misinformation susceptibility, health behaviors and vaccine hesitancy. Future cohort studies should include previous vaccine behavior as a potential confounder in their multivariable models to establish a more valid association between online health misinformation, health behaviors and vaccine hesitancy. Moreover, our data were collected in September 2025, and, therefore, the possibility of seasonal or situational bias cannot be excluded. This period coincides with the onset of autumn, during which public health authorities typically initiate vaccination campaigns to promote influenza immunization. Such contextual factors may have influenced participants’ attitudes or responses and should be considered when interpreting the findings. Moreover, to ensure that all participants had at least a minimal level of exposure to online misinformation, we included individuals who reported ≥30 min of daily web or social media use. As there are currently no established thresholds for what constitutes a meaningful level of exposure to misinformation, this criterion is necessarily arbitrary. Future research employing alternative or multiple exposure thresholds could provide valuable insights and help advance this area of research. Our study included only Greek-speaking participants; therefore, selection bias is likely, as individuals from other countries may hold different perspectives regarding the association between susceptibility to online health misinformation, health behaviors, and vaccine hesitancy. We should also recognize that although we adjusted our multivariable models for several confounding variables, unmeasured confounding is still probable in our study. For instance, we measured digital literacy with only one self-reported item as well as online usage. Measurement of these variables with valid scales such as the eHealth Literacy Scale and the Internet Skills Scale could improve our knowledge on this research topic. Finally, our cross-sectional data reflects only a single moment and cannot capture how online health misinformation susceptibility, health behaviors, and vaccine hesitancy change, evolve, or fluctuate over time. For instance, factors like time of year or weather can influence these variables.

## 5. Conclusions

The present study shows that susceptibility to online health-related misinformation is an individual-level cognitive vulnerability challenge with measurable consequences for health behaviors and vaccine confidence. The associations identified between misinformation susceptibility, maladaptive health behaviors, and vaccine hesitancy underscore the need to conceptualize health misinformation as a risk factor of unhealthy behaviors. However, it should be noted that susceptibility to online health misinformation demonstrated only a weak association with health behaviors and vaccine hesitancy. Additionally, in our study, online health misinformation susceptibility accounted for only a small proportion of the variance in these outcomes. This highlights the need to identify additional factors that may better explain suboptimal health behaviors and vaccine hesitancy within the general population. Moreover, we performed our study only in one country, and thus our findings reflect limited generalizability to the Greek population. In this context, there is an urgent need for longitudinal studies in different countries and cultural settings to further improve our knowledge regarding the association between online health misinformation susceptibility, health behaviors, and vaccine hesitancy.

From a policy standpoint, these findings call for the formal integration of misinformation mitigation into national public health strategies, vaccination policies, and pandemic preparedness frameworks. Governments and public health authorities should establish regulatory and governance mechanisms that promote accountability and transparency in digital health information ecosystems, including structured collaboration with social media platforms to curb the spread of demonstrably false or misleading health content.

## Figures and Tables

**Table 1:** Demographic characteristics of the study sample (n = 402).

Characteristics	N	%
Gender		
Males	94	23.4
Females	308	76.6
Age ^a^	48.74	9.52
Educational level		
High school	40	10.0
University	382	90.0
Financial status ^a^	5.60	1.45
Digital literacy ^a^	7.49	1.90
Daily time on web/social media (h) ^a^	3.00	2.36

^a^ mean, standard deviation.

**Table 2:** Descriptive statistics for the study scales (n = 402).

Scale	Mean	Standard Deviation	Median	Interquartile Range	Range
Health-Related Online Misinformation Susceptibility Scale	22.28	6.82	21	8	9 to 45
Health Behavior Inventory–Short Form					
Diet	2.94	1.37	2.67	2	1 to 7
Proper use of health care resources	2.64	1.49	2.33	2.33	1 to 7
Anger and stress	4.10	1.33	4.33	1.67	1 to 7
Substance use	1.58	0.89	1	1	1 to 7
Vaccine Hesitancy Scale					
Lack of confidence	3.92	0.78	4	1.29	1 to 5
Risk perception	3.06	0.90	3	1.33	1 to 5

**Table 3:** Pearson’s correlation coefficients for the study scales (n = 402).

Scale	2	3	4	5	6	7
1. Health-Related Online Misinformation Susceptibility Scale	0.144 **	0.012	0.147 **	0.084	0.119 *	0.199 **
Health Behavior Inventory–Short Form						
2. Diet		0.457 **	0.041	0.085	0.084	0.098 *
3. Proper use of health care resources			0.082	0.100 *	0.188 **	0.096
4. Anger and stress				0.131 **	0.023	0.093
5. Substance use					0.019	0.048
Vaccine Hesitancy Scale						
6. Lack of confidence						0.288 **
7. Risk perception						

* *p*-value < 0.05. ** *p*-value < 0.01.

**Table 4:** Linear regression models with score on the Health-Related Online Misinformation Susceptibility Scale as the independent variable and scores on the four factors of the Health Behavior Inventory–Short Form as the dependent variables (n = 402).

Dependent Variables	Univariate Models			Multivariable Model ^a^		
	Unadjusted Coefficient Beta	95% CI for Beta	*p*-Value	Adjusted Coefficient Beta	95% CI for Beta	*p*-Value
Diet ^b^	0.029	0.009 to 0.049	0.004	0.026	0.006 to 0.046	0.010
Proper use of health care resources ^c^	0.003	−0.019 to 0.024	0.803	0.000	−0.022 to 0.021	0.984
Anger and stress ^d^	0.029	0.010 to 0.048	0.003	0.033	0.013 to 0.052	0.001
Substance use ^e^	0.011	−0.002 to 0.024	0.092	0.007	−0.006 to 0.020	0.264

^a^ Models are adjusted for gender, age, educational level, financial status, digital literacy, and daily time on web/social media. ^b^ R^2^ for the final multivariable model = 4.0%; *p*-value for ANOVA = 0.002; *p*-value for Breusch–Pagan test = 0.820; Variance Inflation Factor = 1.068. ^c^ R^2^ for the final multivariable model = 4.1%; *p*-value for ANOVA < 0.001; *p*-value for Breusch–Pagan test = 0.380; Variance Inflation Factor = 1.068. ^d^ R^2^ for the final multivariable model = 5.2%; *p*-value for ANOVA < 0.001; *p*-value for Breusch–Pagan test = 0.378; Variance Inflation Factor = 1.068. ^e^ R^2^ for the final multivariable model = 4.5%; *p*-value for ANOVA = 0.001; *p*-value for Breusch–Pagan test = 0.944; Variance Inflation Factor = 1.068. CI: confidence interval.

**Table 5:** Linear regression models with score on the Health-Related Online Misinformation Susceptibility Scale as the independent variable and scores on the two factors of the Vaccine Hesitancy Scale as the dependent variables (n = 402).

Dependent Variables	Univariate Models			Multivariable Model ^a^		
	Unadjusted Coefficient Beta	95% CI for Beta	*p*-Value	Adjusted Coefficient Beta	95% CI for Beta	*p*-Value
Lack of confidence ^b^	0.014	0.003 to 0.025	0.017	0.016	0.005 to 0.028	0.006
Risk perception ^c^	0.026	0.013 to 0.039	<0.001	0.023	0.010 to 0.036	0.001

^a^ Models are adjusted for gender, age, educational level, financial status, digital literacy, and daily time on web/social media. ^b^ R^2^ for the final multivariable model = 3.5%; *p*-value for ANOVA = 0.004; *p*-value for Breusch–Pagan test = 0.235; Variance Inflation Factor = 1.068. ^c^ R^2^ for the final multivariable model = 6.1%; *p*-value for ANOVA < 0.001; *p*-value for Breusch–Pagan test = 0.382; Variance Inflation Factor = 1.068. CI: confidence interval.

## Data Availability

The data used in this study are openly available in Figshare at https://doi.org/10.6084/m9.figshare.30675122 (accessed on 8 January 2026).
